# Knowledge, attitudes and practices of hospital-based staff regarding physical activity at a private hospital in Johannesburg

**DOI:** 10.4102/safp.v63i1.5131

**Published:** 2021-01-12

**Authors:** Yurisha Ramautar, Boikhutso Tlou, Thembelihle P. Dlungwane

**Affiliations:** 1 School of Nursing and Public Health, Discipline of Public Health Medicine, University of KwaZulu-Natal, Durban, South Africa

**Keywords:** knowledge, hospital-based staff, physical activity, practice, attitude

## Abstract

**Background:**

Physical activity has been established as an important component to incorporate into a healthy lifestyle. Hospital-based staff are also threatened by the risks of sedentary lifestyles, despite their association with a healthcare environment. The aim of this study was to determine the knowledge, attitudes and practices of private hospital-based staff regarding physical activity in Johannesburg.

**Methods:**

A cross-sectional study was conducted. Data were collected using a self-administered questionnaire. Data were analysed with a combination of descriptive and inferential statistics. A *p*-value less than 0.05 was deemed statistically significant.

**Results:**

A total number of 217 participants responded to the questionnaire. The majority of participants (*n* = 179; 82.49%) displayed excellent knowledge of physical activity, had a good attitude towards physical activity (*n* = 157; 72.35%) and displayed satisfactory practices (*n* = 137; 63.13%). Participants with the highest level of education had better mean knowledge, attitude and practice scores as opposed to those with lower levels of education. There was a statistically significant difference amongst staff categories in terms of knowledge (*p* = 0.004) and practice scores (*p* = 0.031). In addition, there was a statistically significant difference amongst different levels of education in terms of knowledge (*p* = 0.000), attitude (*p* = 0.02) and practice scores (*p* = 0.004).

**Conclusion:**

Staff members who participated in the study displayed only satisfactory physical activity practices. The hospital’s employee wellness programme should establish appropriate strategies to improve staff practices of physical activity in order to promote health.

## Introduction

The World Health Organization (WHO) has ranked physical inactivity as one of the leading risk factors for non-communicable diseases,^1^ and estimates that 31% of the global healthy population does not fulfil the recommended physical activity requirement.^1,2^ Regular and moderate-intensity physical activity offers significant health benefits,^3^ including reduced disability and mortality, improvement in quality of life and enhanced psychological well-being.^4^ Failure to engage in the required amount of physical activity places one at risk of a variety of chronic and lifestyle-related diseases such as diabetes mellitus type II, cardiovascular diseases, cancer, cerebrovascular accidents, hypertension, obesity and osteoarthritis.^3^ Physical inactivity contributes to increased medical expenditure and social burden as a result of the prevalence of disability in the adult workforce.^4^

The American College of Sports Medicine and the American Heart Association state that:^5,6,7,8^

[*I*]n order to promote and maintain health, all healthy adults aged 18 to 65 years need to engage in moderate-intensity aerobic (endurance) physical activity for a minimum of 30 min on 5 days each week, or vigorous-intensity aerobic physical activity for a minimum of 20 min on 3 days each week. (pp. 1083–1084)

Recommendations and guidelines regarding the perceived benefits of physical activity have evolved drastically over the years, which includes the personal and environmental characteristics of the activity.^9^ Despite efforts aimed at educating the public on the importance of physical activity, physical inactivity remains a public health concern.^7,10^ This holds true especially for developing countries such as South Africa, which has a rapidly emerging non-communicable disease epidemic.^11,12^ Determinants of physical activity are multidimensional and act as either barriers or facilitators to health.^7^ These include psychological, musculoskeletal, social and environmental components.^13^

Staff members working in a healthcare environment are viewed as ambassadors of health by their families and the communities they live in, irrespective of whether their roles are medical or non-medical. However, despite their professional status or association with a healthcare environment, hospital staff are also at an increased risk of sedentary lifestyle.^14,15,16^ Clinical staff at hospitals should possess good knowledge of the benefits of physical activity, in order to provide effective counselling to patients with regard to improving physical activity levels.^17^ Studies also support the fact that clinical staff who adopt good personal practices of physical activity are more likely to counsel their patients on the importance of being physically active.^17,18^ Individuals who work in a hospital are assumed to have better knowledge than others of the benefits of physical activity. Their practices are also assumed to model those of a healthy lifestyle.

Studies conducted at public hospitals in South Africa reported that hospital staff displayed low levels of physical activity.^16,19^ Research regarding healthcare workers and physical activity in the South African context have focused on the public sector, where resources are scarce and incentives to promote health-seeking behaviours few. On the contrary, the private healthcare sector is well funded and has well-developed employee wellness programmes. However, there are few or no studies that have explored physical activities in the latter context in South Africa. This study aims to assess the knowledge, attitudes and practice (KAP) of private hospital-based staff regarding physical activity in Johannesburg.

## Methods

### Study design

A cross-sectional study was conducted.

### Study setting

The study was conducted at a private hospital in Johannesburg. The hospital was once a private community clinic and has developed over the years to become a leading private medical facility with subspecialties. The hospital offers several specialist services, such as neurology, pulmonology, orthopaedics, cardiology, and adult and paediatric cardiothoracic surgery. The hospital is also home to the centre of excellence in paediatrics and has one of the leading emergency trauma centres on the African continent.

### Study population and sampling

The hospital had approximately 460 staff of different categories, namely, administrative, allied health, support staff, and medical and nursing practitioners. The study used the Krejcie and Morgan (1970)^20^ table to estimate the study sample size of 210. Convenience sampling was used: all staff on duty on the day and night shifts during the time of data collection were approached.

### Tool and data collection

Self-administered questionnaires were used for this study. The questionnaire was used and validated in a previous study conducted to determine the KAP of healthcare workers regarding healthy lifestyles,^21^ with an overall Cronbach’s alpha of 0.83. The questionnaire was divided into four sections: (1) sociodemographic characteristics, (2) staff member’s knowledge of physical activity, its benefits and the minimum amount of physical activity required to maintain one’s health, (3) attitude towards physical activity and (4) practices of the staff member regarding physical activity.

At the time of data collection, the researcher and a research assistant visited various departments within the hospital to explain the purpose of the study to the participants. The research assistant then distributed questionnaires, which were collected from the participants once they were completed. The questionnaire was pretested with 10 staff members who were not included in the main study.

### Data analysis

The knowledge score was obtained by assigning a point of 1 for each question answered correctly, and a 0 for each incorrectly answered question. Knowledge responses were aggregated and evaluated. Participants who had > 80% correct responses were considered to have excellent knowledge, and those who had 60% – 80% correct responses were regarded as having good knowledge. The participants with scores of 50% – 59% and < 50% correct responses were considered to have some knowledge and poor knowledge, respectively.

A five-level Likert scale was used to collect data on attitudes and practices. The participants were asked to rate the levels of attitude and practice from 1 to 5: 5 = strongly agree, 4 = agree, 3 = neither agree nor disagree, 2 = disagree and 1 = strongly disagree. The responses were aggregated and evaluated. A score less than 60% was considered as a poor attitude and unsatisfactory for practice.

Data were first captured onto a Microsoft Excel spreadsheet and then imported into the statistical software package SAS for data analysis. Frequency distribution tables were used to summarise the demographic characteristics for the study participants. Furthermore, an analysis of variance was performed to compare the mean KAP scores to the staff category, highest level of education and age category. An independent sample *t*-test was also performed to assess the mean KAP scores by gender and participant’s engagement in physical activity. A *p*-value of less than 0.05 was deemed as statistically significant.

### Ethical consideration

The study protocol was submitted by the researcher to the Biomedical Research and Ethics Committee (BREC) of the University of KwaZulu-Natal, and ethics approval was granted (BREC reference number BE485/16). Approval to conduct research at the facility was granted (reference number UNIV-2016-0070). Written informed consent was obtained from the individual participants who agreed to participate in the study and who met the inclusion criteria. The study participants’ identities were kept confidential by using codes as identifiers on the questionnaires. In addition, password-protected files and folders were used to keep the records, whilst completed questionnaires were locked in office drawers.

## Results

A total of 231 questionnaires were administered, of which 217 were adequately completed, yielding a response rate of 90.4%. Most of the participants were female (*n* = 161; 74.19%), aged between 30 and 39 years of age (*n* = 83; 38.25%), had a diploma (*n* = 90; 41.48%) and were in the administrative staff category (*n* = 78; 35.95%). The majority of participants were reported to have engaged in physical activity (*n* = 150; 69.12%) (Table 1).

**TABLE 1:** Demographic characteristics of participants.†

Demographic characteristic	*n*	%
**Gender**		
Female	161	74.19
Male	56	25.81
**Age**		
20–29	52	23.96
30–39	83	38.25
40–49	42	19.36
50–59	27	12.44
60+	13	5.99
**Staff category**		
Administrative	78	35.95
Allied health	13	5.99
General/support staff	48	22.12
Medical	26	11.98
Nursing	52	23.96
**Highest level of education**		
Secondary school	28	12.90
Diploma	90	41.48
Bachelor’s degree	24	11.06
Postgraduate degree	75	34.56
**Engage in physical activity†**		
Yes	150	69.12
No	67	30.88

† , *n* = 217.

The majority of participants (82.49%) displayed excellent knowledge of physical activity, and 72.35% had a good attitude towards physical activity. However, 63.13% revealed only satisfactory practices (Table 2).

**TABLE 2:** Knowledge, attitudes and practices of participants.†

Characteristic	*n*	%
**Knowledge**		
Excellent	179	82.49
Good	38	17.51
**Attitude**		
Good	157	72.35
Poor	60	27.65
**Practice**		
Satisfactory	137	63.13
Unsatisfactory	80	36.87

† , *n* = 217.

About 98% of the study participants indicated that regular physical activity improves health. In addition, more than 90% of the participants indicated that regular physical activity decreases cholesterol and blood pressure, prevents heart disease and maintains one’s body weight (Table 3).

**TABLE 3:** Participants’ knowledge of physical activity.†

Statement	No. of participants who agreed with statement	
	*n*	%
Regular physical activity can prevent heart disease.	212	97.70
Regular physical activity can help improve health.	214	98.62
Regular physical activity doesn’t increase the risk of developing depression and anxiety.	189	87.10
Regular physical activity can cause a drastic drop in blood pressure.	177	81.57
Regular physical activity helps maintain one’s body weight.	212	97.70
Regular physical activity doesn’t increase the risk of developing diabetes type 2.	178	82.03
Regular physical activity decreased cholesterol and blood pressure.	202	93.09
Regular physical activity doesn’t increase the risk of contracting HIV/AIDS.	204	94.01
Regular physical activity reduces the risk of getting osteoporosis.	162	74.65
One should engage in moderate-intensity exercise for 30 min on 5 days per week, to maintain good health (e.g. brisk walking, swimming, volleyball)	197	90.78
One should engage in vigorous-intensity exercises for 20 min on 3 days per week, to maintain good health (e.g. running, soccer)	170	78.34

HIV/AIDS, human immunodeficiency virus/acquired immune deficiency syndrome.

† , *n* = 217.

More than 90% of the participants indicated that exercise provides more benefits towards their health and that they were willing to engage in more physical activity to stay physically healthy. In addition, about 67% of the participants felt that they would engage in physical activity if their workplace had a gymnasium and a wellness programme. Approximately 45% of the study participants indicated their displeasure with their current body weight (Table 4).

**TABLE 4:** Participants’ attitudes towards physical activity.

Statement	Strongly agree (%)	Agree (%)	Neither agree nor disagree (%)	Disagree (%)	Strongly disagree (%)
Exercise provides many benefits, no matter what age we start.	69.59	28.57	0.92	0.00	0.92
It is important for me to me to be physically healthy.	72.81	24.42	1.84	0.46	0.47
My family history will determine what disease I get, so it doesn’t matter if I exercise or not.	4.61	9.22	14.29	35.48	36.40
I would engage in more physical activity if I had more time.	45.62	37.33	8.29	5.53	3.23
There are many more important things in life than being physically active.	6.45	9.22	17.05	38.71	28.57
I would engage in physical activity if my workplace had facilities, e.g., a gymnasium, wellness programme.	43.32	23.96	11.52	14.75	6.45
I would engage in more physical activity if I had more motivation from my colleagues.	21.66	18.89	16.59	32.26	10.60
A reward programme at work would be an incentive for me to be more physically active.	25.35	24.88	17.05	24.88	7.84
We should not rely on our workplace to provide us with incentive to exercise.	24.42	36.87	20.28	11.06	7.37
Fat people are healthier people.	2.30	0.92	16.13	20.28	60.37
I am happy with my current body weight.	17.05	28.11	16.13	23.96	14.75
Exercising is expensive, and only wealthy people can afford to do it.	5.53	6.45	16.13	31.34	40.55

More than 55% of the study participants indicated that they engaged in the right amount of physical activity and that they intended to start doing more physical activity in the next 6 months. About 53% of the study participants indicated that they engaged in exercise for pleasure as well, instead of only for the health benefits. In addition, 33.17% of participants highlighted that they got all the exercise they needed from just conducting their normal activities (Table 5).

**TABLE 5:** Participants’ physical activity practices.

Statement	Strongly agree (%)	Agree (%)	Neither agree nor disagree (%)	Disagree (%)	Strongly disagree (%)
I engage in the right amount of physical activity.	29.03	30.41	17.05	18.89	4.62
I have a medical condition that prevents me from engaging in physical activity.	5.99	10.14	11.52	35.48	36.87
I engage in exercise for pleasure, not only for health benefits.	24.42	28.11	19.35	20.28	7.84
I intend to start doing more physical activity in the next 6 months.	23.96	34.10	24.42	10.14	7.38
I get all the exercise I need from just doing normal activities.	13.82	19.35	20.28	34.10	12.45
I engage in vigorous intensity sports for 20 min, 3–5 times a week (e.g. running, soccer).	17.51	22.12	11.06	31.80	17.51
I engage in moderate-intensity sports for 30 min, more than 5 times a week (e.g. brisk walking, dancing).	24.42	24.42	10.60	27.65	12.91

There was a statistically significant difference amongst staff categories in terms of knowledge (*F* = 3.93, *df* = 4, *p* = 0.0043) and practice (*F* = 2.72, *df* = 4, *p* = 0.0307) scores. Allied healthcare workers had higher knowledge and practice scores as opposed to other staff categories. In addition to that, there was a statistically significant difference amongst the different levels of education in terms of knowledge (*F* = 6.14, *df* = 3, *p* = 0.0005), attitude (*F* = 3.36, *df* = 3, *p* = 0.0198) and practice (*F* = 4.57, *df* = 3, *p* = 0.004) scores. Participants with the highest level of education (tertiary education and above) had better mean KAP scores as opposed to those with lower levels of education. Moreover, participants who engaged in physical activity had significantly higher knowledge (*t* = –2.52, *df* = 215, *p* = 0.0124), attitude (*t* = –3, *df* = 215, *p* = 0.003) and practice (*t* = –8.04, *df* = 215, *p* < 0.0001) scores as compared with those who did not engage in any physical activity. In addition, this study found that females were more knowledgeable (*t* = 2.03, *df* = 215, *p* = 0.0432) as compared with men. Age was not significantly associated with KAP (Table 6).

**TABLE 6:** Differences in knowledge, attitudes and practices and respondent characteristics.†

Participant characteristic	Knowledge			Attitude		Practice	
	*n*	Mean	*p*	Mean	*p*	Mean	*p*
**Gender**							
Female	161	16.71	0.043	3.62	0.426	3.56	0.385
Male	56	16.17		3.57		3.63	
**Age**							
20–29	52	16.40	0.606	3.54	0.297	3.63	0.368
30–39	83	16.35		3.66		3.63	
40–49	42	16.79		3.54		3.48	
50–59	27	16.81		3.69		3.45	
60+	13	17.00		3.53		3.58	
**Staff category**							
Administrative	78	16.41	0.004	3.57	0.187	3.56	0.031
Allied health	13	18.08		3.80		3.79	
General/support staff	48	16.00		3.56		3.51	
Medical	26	17.31		3.73		3.83	
Nursing	52	16.48		3.59		3.27	
**Highest level of education**							
Secondary school	28	15.91	0.001	3.52	0.020	3.53	0.004
Diploma	90	16.57		3.59		3.48	
Bachelor’s degree	24	17.36		3.79		3.78	
Postgraduate degree	75	17.50		3.71		3.84	
**Engage in physical activity** †							
Yes	150	16.77	0.012	3.66	0.003	3.75	< 0.001
No	67	16.03		3.48		3.19	

† , *n* = 217.

## Discussion

The results of the study reveal that the participants displayed excellent knowledge of physical activity and a good attitude towards physical activity. However, their practices were only satisfactory. This is in contrast with the findings of a study that was conducted amongst hospital-based staff who were employed in the public health service in South Africa, which concluded that the majority of staff did not engage in regular physical activity.^2,10^ This could be attributed to the lack of free time, the working hours and the environment, which is physically taxing and which could influence one’s willingness to engage in physical activity.

The allied health and medical practitioners in this study displayed the best knowledge of physical activity. This is consistent with the results of the study that was conducted in Pretoria, South Africa, which concluded that medical staff had better knowledge of physical activity than non-medical staff.^2^ Participants with postgraduate degrees had the highest levels of knowledge and the best physical activity practices, whilst those with secondary schooling as their highest level of education displayed the lowest scores in terms of knowledge and practices. These findings suggest that the participants had greater levels of health literacy and education, which may facilitate healthy lifestyle behaviours. In addition, there was a statistically significant difference amongst the different levels of education in terms of knowledge (*F* = 6.14, *df* = 3, *p* = 0.0005), attitude (*F* = 3.36, *df* = 3, *p* = 0.0198) and practice (*F* = 4.57, *df* = 3, *p* = 0.004) scores.

Participants with the highest level of education had better mean KAP scores as opposed to those with lower levels of education. The findings of this study concur with a study conducted amongst healthcare workers in Sweden, which reported that participants with a higher level of education had better knowledge of the benefits of physical activity. However, the study in Sweden found that having better knowledge of the benefits of physical activity did not translate into the individuals having better practices of physical activity.^22^

Gender roles affect regular exercise behaviour because of the difference in social obligations between men and women. With regard to knowledge of physical activity, women achieved slightly better scores than men in this study, but there was no significant difference found between genders with regard to attitudes and practices of physical activity. This might be because gender roles have changed, which has resulted in both men and women participating in most physical activities. These results are in line with a study conducted in Iran amongst female physicians, nurses and midwives, in which all the participants were aware of the role of physical activity on overall well-being.^23^ However, the literature on the relationship between gender and physical activity varies. Some studies have reported that there is no statistical relationship between gender and participation in physical activity, whilst other studies have reported one gender group to be more active than the other.^2,7,19,24,25,26,27^ Further research should be conducted to investigate the relationship between gender and attitudes and practices regarding physical activity.

Most of the participants (57.6%) who indicated that they engaged in physical activity were aware of the minimum requirements for vigorous-intensity physical activity, whilst 62.21% were aware of the minimum requirements for moderate-intensity physical activity. Moreover, participants who engaged in physical activity had significantly higher knowledge (*t* = –2.52, *df* = 215, *p* = 0.0124), attitude (*t* = –3, *df* = 215, *p* = 0.003) and practice (*t* = –8.04, *df* = 215, *p* < 0.0001) scores as compared with those who did not engage in any physical activities. These results differ from the results obtained in a study in Brazil amongst nurses, physicians and other healthcare workers, which revealed that all three groups lacked knowledge of the recommended minimum requirements for physical activity.^17^

Overall, the scores reveal that the staff displayed positive attitudes towards physical activity. Most of the participants in the current study strongly agreed that it was important for them to be physically active. Those individuals who engaged in physical activity had a more positive attitude towards physical activity than those who indicated that they did not engage in physical activity. A study in Spain found that physicians and nurses perceived physical activity as ‘very important’. However, they also reported several barriers to improving their own personal levels of activity.^28^ These barriers included lack of time and isolation from physical activity facilities in their communities.^28^ Similar results were found in a study conducted in the United States of America amongst physician’s assistants and nursing practitioners, who displayed poor attitudes towards physical activity.^29^ Unsafe neighbourhoods influenced the attitudes of the physician’s assistants and nursing practitioners towards physical activity.^29^ Participants in this study indicated that they would engage in more physical activity if they had more time and if their workplace had facilities such as a gym and wellness programme. Workplace wellness programmes should include physical activity sessions within the institution.

Medical staff in this study scored the highest in terms of their practices of physical activity, whilst nurses scored the lowest. These results are supported by other studies, which reported poor levels of physical activity amongst nurses in KwaZulu-Natal and the Western Cape.^30,31,32^

These findings may suggest that health-promoting strategies should be adjusted for the category of staff that have reported low levels of physical activity. Sixty per cent of the participants in this study felt that they engaged in the right amount of physical activity. However, only 39.63% of the participants engaged in the required amount of vigorous-intensity physical activity, whilst 48.8% of the participants engaged in the required amount of moderate-intensity physical activity. In addition, 33% of the participants highlighted that they got all the exercise they needed from just conducting their normal activities. Studies done among healthcare workers in Iran, Nigeria and South Africa, reported poor practices of physical activity.^18,19,23^ The current study findings reveal that the participants reported having satisfactory practices of physical activity, with ample room for improvement to good or excellent practices. However, the intensity and duration were suboptimal according to the recommendations and may not accrue to any significant health benefit.^5,6,7^ This could be because of the absence of appropriate strategies to enhance staff participation in physical activity as part of the hospital’s employee wellness programme.

### Study limitations

This survey was based on self-reporting by participants and therefore was subject to social desirability bias. The study was conducted in one private health institution, and the results may therefore not be generalisable to hospital-based staff working in other private health institutions.

## Conclusion

The participants displayed an excellent level of knowledge and a good attitude towards physical activity. However, the staff displayed only satisfactory practices of physical activity. The hospital’s employee wellness programme should therefore establish appropriate strategies that enhance the staff’s physical activity practice to promote health. These strategies should include advocacy for facilities such as a gym on site or exercise classes to be established within the workplace. In addition, time should be scheduled to allow staff to participate in these physical activities.
